# Fibroblast-epithelial cell interactions drive epithelial-mesenchymal transition differently in cells from normal and COPD patients

**DOI:** 10.1186/s12931-015-0232-4

**Published:** 2015-06-18

**Authors:** Michiyoshi Nishioka, Narayanan Venkatesan, Kevin Dessalle, Andrea Mogas, Shigenori Kyoh, Ting-Yu Lin, Parameswaran Nair, Carolyn J. Baglole, David H. Eidelman, Mara S. Ludwig, Qutayba Hamid

**Affiliations:** Meakins-Christie Laboratories, Research Institute-McGill University Health Centre, 1001 Decarie Boulevard, Block E, Montreal, QC H4A 3J1 Canada; Department of Thoracic Medicine, Chang Gung Memorial Hospital, College of Medicine, Chang Gung University, Taipei, Taiwan; Department of Medicine, McMaster University, Hamilton, ON Canada

**Keywords:** Cigarette smoking, COPD, EMT, Epithelial cells, Fibroblasts

## Abstract

**Background:**

Epithelial-to-mesenchymal transition (EMT), which involves changes in cellular morphology of highly polarized epithelial cells and the gain of mesenchymal cell phenotype with migratory and invasive capacities, is implicated in smoking-related chronic obstructive pulmonary disease (COPD). However, the interactions of fibroblasts and epithelial cells and the participation of fibroblasts in the EMT processes in COPD are poorly understood. Here, we investigated the hypothesis that EMT is active in human bronchial epithelial (HBE) cells of COPD patients, and that mediators secreted by lung fibroblasts from COPD patients induce EMT.

**Methods:**

Primary HBE cells from normal subjects and COPD patients were purchased from LONZA. HLFs were derived from resected lung obtained from normal (N) and COPD (D) subjects and their conditioned medium (CM) was collected after 2-day culture in serum-free medium. The expression of epithelial and mesenchymal markers as well as EMT-related transcription factors in lung biopsies, and in HBE cells following stimulation with CM from both normal human lung fibroblasts (NHLF) and COPD human lung fibroblasts (DHLF) was evaluated by immunohistochemistry, qRT-PCR and western blot.

**Results:**

Basal mRNA expression of mesenchymal markers and EMT-related transcription factors were increased in DHBE cells compared to normal human bronchial epithelial cells (NHBE) cells as well as in COPD lungs. CM from NHLF significantly induced vimentin expression in both NHBE and COPD human bronchial epithelial cells (DHBE) cells, but only increased N-cadherin expression in DHBE cells. CM from NHLF significantly induced Twist1 and Twist2 expression in NHBE cells and increased Snai2 (Slug) expression in DHBE cells. While CM from NHLF had no effect on such EMT markers, CM from DHLF significantly increased the protein expression of E-cadherin and vimentin in NHBE cells compared to control. N-cadherin expression was upregulated to a greater degree in NHBE cells than DHBE cells. Only CM from DHLF significantly increased E-/N-cadherin ratio in DHBE cells.

**Conclusions:**

Our results suggest that DHBE cells have partially undergone EMT under baseline conditions. DHLF-CM promoted EMT in NHBE, suggesting that interactions between fibroblast and epithelial cells may play an important role in the EMT process in COPD.

## Background

Chronic obstructive pulmonary disease (COPD) is characterized by airflow limitation that is progressive and not fully reversible. Cigarette smoking is the main risk factor for COPD contributing to structural changes in the airways during COPD progression [1]. Airway remodeling events in COPD include goblet cell hyperplasia and mucus hypersecretion, increased smooth muscle mass, and airway wall fibrosis. Thickening of the airway wall and peribronchiolar fibrosis play an important role in the development of symptoms associated with loss of lung function in COPD. An important characteristic of the fibrotic response is the increased deposition of extracellular matrix (ECM) proteins, particularly collagen types I and III, fibronectin and proteoglycans.

One of the mechanisms now implicated in remodeling events associated with COPD pathogenesis is that fibroblastic cells arise from local conversion of epithelial cells by epithelial-mesenchymal transition (EMT) induced by cigarette smoking [2]. During EMT, epithelial cells lose their typical cell-cell junctions and cell polarity and acquire a more motile, mesenchymal phenotype [3]. The most common transformation occurs from epithelial cells to fibroblasts, which subsequently make the transition to myofibroblasts. EMT is mainly characterized by the loss of epithelial markers such as cytokeratins, tight junction proteins and E-cadherin, the acquisition of mesenchymal markers such as vimentin and N-cadherin, and increased expression of the transcription factors Snail, Twist and Zeb [3].

EMT has been recently shown to be important in COPD [2]. Sohal et al. reported that, in smokers and COPD patients, cells in the fragmented reticular basement membrane (Rbm) are double-positive for vimentin and cytokeratins, suggestive of EMT [4]. The presence of cells expressing S100A4 and vimentin in the basal layer of the epithelium in smokers and COPD patients also indicates the contribution of smoke exposure to EMT process [5]. Human bronchial epithelial cells (HBECs) and A549 cells undergo EMT *in vitro* after treatment with cigarette smoke condensate [6–8] further strengthening the rationale that EMT is a contributing factor in remodeling events of COPD.

Interaction between lung structural cells, particularly epithelial cells and fibroblasts, may be key in driving the EMT process in COPD. Bronchial epithelial cells are the first anatomical barrier to noxious cigarette smoke particles and are involved in the initiation of airway remodeling through the production of proinflammatory mediators, ECM protein, growth factors and matrix metalloproteinases [9]. Supernatants from bronchial epithelial cell cultures contain factors which both stimulate and inhibit fibroblast proliferation [10]. Fibroblasts are also important in regulating ECM turnover and epithelial cell differentiation via growth factor secretion and mesenchymal-epithelial cell interactions [11]. However, the interactions of fibroblasts and epithelial cells and the participation of fibroblasts in the EMT process remain poorly understood in COPD.

In this study, we hypothesized that EMT is active in bronchial epithelial cells of patients with COPD, and that mediators secreted by COPD lung fibroblasts could induce EMT. We therefore investigated the EMT process in bronchial epithelial cells of COPD patients, together with the effect of mediators secreted by human lung fibroblasts (HLF) from normal and COPD subjects on the expression of epithelial and mesenchymal markers in human bronchial epithelial (HBE) cells.

## Methods

### Epithelial cell culture

Primary human bronchial epithelial cells from normal subjects (NHBE) and COPD patients (DHBE) were purchased from Lonza (Walkersville, MD) and were maintained in serum-free bronchial epithelial cell growth medium (BEGM, Lonza) supplemented with a bullet kit containing bovine pituitary extract, insulin, hydrocortisone, gentamicin/amphotericin, retinoic acid, transferrin, epinephrine and human epithelial growth factor (hEGF) (Lonza). NHBE and DHBE cells were used before passage 6.

### Fibroblast cell culture and collection of conditioned media (CM)

Lung tissue was obtained from individuals undergoing lung resection surgery for suspected lung cancer at McMaster University. Recruited individuals included those with COPD as well as never-smokers without COPD (controls). This study was approved by the Research Ethics Board of St Joseph’s Healthcare Hamilton and all patients gave written informed consent. Primary lung fibroblasts were cultured as previously described [12, 13] from parenchymal lung tissue. Only tissue from cancer-free regions was used for the derivation of fibroblasts. Prior to experimentation, fibroblasts were characterized based on morphology, vimentin expression and absence of cytokeratin (epithelial cell marker), desmin (muscle cell marker) and α-smooth muscle actin (α-SMA; myofibroblast marker) [12, 13]. All fibroblasts used in this study had a typical fibroblast morphology (flat, elongate with oval nuclei) and expressed vimentin; no staining was observed for cytokeratin or desmin, Following characterization, cells were expanded and either frozen in liquid nitrogen or maintained in culture. For experimentation, primary human lung fibroblasts from normal subjects (NHLF) and COPD patients (DHLF) were maintained in minimum essential medium (MEM) supplemented with 2 mM L-glutamine (Life Technologies, Burlington, ON), 10 % fetal bovine serum, and antibiotics (50 μg/ml streptomycin and 50 U/ml penicillin). Cells were maintained at 37 °C and incubated in humidified 5 % CO_2_ atmosphere. Fibroblasts were used before passage 14.

For experimentation, fibroblasts (3 × 10^5^ cells per flask) were plated in T-75 flasks in 10 % FBS MEM and allowed to reach 80 % confluence. The cultures were then rinsed twice and cultured in serum-free growth medium consisted of BEGM and Dulbecco’s Modified Eagle’s Medium (DMEM) and CM was collected 48 h later.

### Treatment of epithelial cells with fibroblast-derived CM

NHBE and DHBE were grown at 37 °C in a humidified 5 % CO_2_ atmosphere. Prior to the experiments, the cells were starved in 1 % BEGM for 24 h and then stimulated with CM derived from NHLF or DHLF for a period of 3 (RNA analysis) or 5 (protein analysis) days. The cells were then harvested for further analysis.

### Protein quantification and immunoblotting

Primary bronchial epithelial cells were lysed in 80 μL of lysis buffer (50 mM Tris–HCl pH 7.5, 1 mM EGTA, 1 mM EDTA, 1 % (v/v) Triton X-100, 1 mM sodium orthovanadate, 5 mM sodium pyrophosphate, 50 mM sodium fluoride, 0.27 M sucrose, 5 mM sodium pyrophosphate decahydrate and protease inhibitors). Protein concentrations were quantified using the BCA Protein Assay Kit (ThermoScientific, Rockford, IL) according to the manufacturer’s instructions. Forty micrograms of protein were boiled and separated on a 10 % Pro-Pure Next Gel with Pro-Pure Running Buffer (Amresco, Solon, OH). After transferring proteins to nitrocellulose, membranes were blocked for 1 h at room temperature in Odyssey Blocking Buffer (Li-Cor Biosciences, Lincoln, NE). Blots were then incubated with a mouse anti-human vimentin antibody (1:200, sc-6260, Santa Cruz Biotechnology, Santa Cruz, CA), a mouse anti-human E-cadherin antibody (1:600, ab1416, Abcam, Cambridge, MA), a rabbit anti-human N-cadherin antibody (1:1000, ab76057, Abcam) or a mouse anti-human GAPDH antibody (1:1500, MAB374, Millipore, Billerica, MA) overnight at 4 °C. Goat anti-mouse IgG (1:15,000, #35518, DyLight™680, Thermo Scientific) antibody or goat anti-rabbit IgG (1:15,000, #35571, DyLight™800, Thermo Scientific) antibody was applied for 1 h in the dark at room temperature (1:15,000). The signal was detected and quantified using a LI-COR Odyssey imaging system (LI-COR Biosciences). All samples were normalized to GAPDH and expressed as a ratio relative to the control sample.

### Measurement of hepatocyte growth factor (HGF)

HGF concentration in cell cultured conditioned medium was measured with human HGF ELISA kit (R&D Systems, Inc. Minneapolis, MN, USA) according to the manufacturer’s protocol. HGF secretion by fibroblasts was expressed in picogram/ml.

### Semi-quantitative RT-PCR

Total RNA was isolated from cultured primary bronchial epithelial cells and purified using the RNeasy Mini Kit (Qiagen, Toronto, Canada), supplemented with the RNase-Free DNase Set (Qiagen). cDNA was obtained using the SuperScript^TM^ III Reverse Transcriptase (Life Technologies), and the absence of DNA contamination was verified by excluding the reverse transcriptase from subsequent PCR reactions. cDNA was subjected to PCR using the Power SYBR Green PCR Master Mix (Applied Biosystems, Foster City, CA) to amplify human transcripts of E-cadherin, N-cadherin, vimentin, Snail1, Snail2, Twist1, Twist2, Zeb1, Zeb2 and GAPDH using primers from Life Technologies. Each set of primers are shown in Table 1. Each PCR reaction was carried out as follows: 15 min at 95 °C, 15 s at 94 °C, 30 s at 60 °C, and 30 s at 72 °C. Each cycle was repeated 40 times following the manufacturer’s recommendations using a 7500 Fast Real-Time PCR System (Applied Biosystems) thermal cycler. Based on the comparative Ct method, gene expression levels were calculated and GAPDH was used as the housekeeping gene. Untreated control samples for each cell line were set to 100 % and the fold change in expression following treatment is represented as mean ± standard error of the mean. Each condition was assessed based on duplicates with *n* = 3 cell lines per group.

**Table 1:** Primers for quantitative real-time RT-PCR analyses of gene transcript expression

Gene	Forward primer	Reverse primer	Size (bp)
E-cadherin	5’-gccgagagctacacgttca-3’	5’-gaccggtgcaatcttcaaa-3’	88
N-cadherin	5’-ctccatgtgccggatagc-3’	5’-cgatttcaccagaagcctctac-3’	92
Vimentin	5’-gtttcccctaaaccgctagg-3’	5’-agcgagagtggcagagga-3’	68
Snail	5’-gctgcaggactctaatccaga-3’	5‘-atctccggaggtgggatg-3’	84
Slug	5’-tggttgcttcaaggacacat-3’	5‘-gttgcagtgagggcaagaa-3’	66
Twist1	5’-aaggcatcactatggactttctct-3’	5‘-gccagtttgatcccagtatttt-3’	96
Twist2	5’-tctgaaacctgaacaacctcag-3’	5’-ctgctgtcccttctctcgac	70
Zeb1	5’-gctaagaactgctgggaggat-3’	5’-atcctgcttcatctgcctga-3’	79-82
Zeb2	5’-aagccagggacagatcagc-3’	5’-ccacactctgtgcatttgaact-3’	74
HGF	5’-cagagggacaaaggaaaagaa-3’	5’-gcaagtgaatggaagtccttta-3’	167
GAPDH	5’-agtcaacggatttggtcgtatt-3’	5’-atgggtggaatcatattggaac-3’	139

### Immunohistochemistry

Paraffin-embedded lung sections were stained with specific antibodies for E-cadherin (a mouse anti-human E-cadherin antibody, 1:300 dilution, ab1416, Abcam, Cambridge, MA) or snail1 (1:250 dilution, a rabbit anti-Snail + Slug, ab85936, Abcam, Cambridge, MA) or N-cadherin (a rabbit polyclonal anti-human N-cadherin antibody, 1:300 dilution, ab18203, Abcam, Cambridge, MA). For negative controls, isotype-matched control antibody was substituted for primary antibody (data not shown). Lung sections were incubated with peroxidase-antiperoxidase complex and developed with diaminobenzidine (DAB) after blocking endogenous peroxidase activity. All sections were counterstained with Gill II hematoxylin, inspected using an Olympus light microscope and digital images were saved. Dark brown color indicates positive immunostaining for a particular antigen expression.

### Statistical analysis

For statistical analyses between two groups, a *t*-test was used. Comparisons among more than two groups were performed by ANOVA, followed by a Tukey post hoc test. A p-value of < 0 .05 was considered to be statistically significant. Data are expressed as mean ± standard error of the mean.

## Results

### Basal expression of EMT markers and EMT-related transcription factors are increased in COPD bronchial epithelial cells and COPD lung tissues

**Fig. 1:**
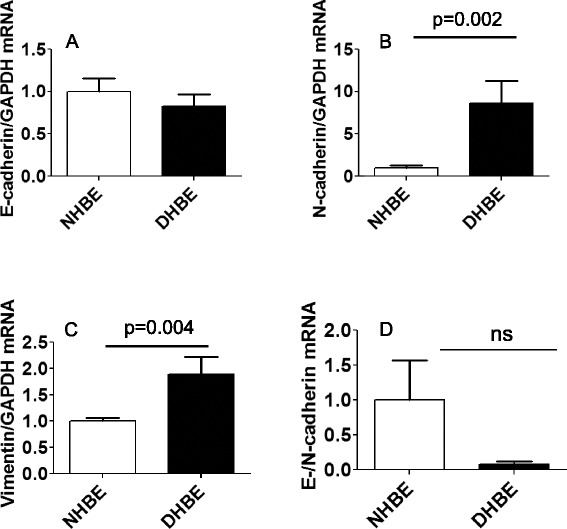
Basal mRNA expression of mesenchymal markers was increased in DHBE cells. Basal mRNA expression of EMT-related markers in non-COPD (NHBE, open bar, *n* = 3) and COPD (DHBE, closed bar, *n* = 3) human bronchial epithelial cells were analyzed by real-time RT-PCR. Results were normalized to NHBE and expressed as mean (SE) of three independent experiments. Data were analysed by Student *t* test, *p < 0.05, compared with NHBE. (**a**) E-cadherin (**b**) N-cadherin (**c**) vimentin (**d**) Ratio of E-/N-cadherin. ns: not significant

To determine the basal expression of EMT-related markers in HBE cells, we examined the mRNA expression of epithelial cells derived from normal subjects (NHBE) and COPD patients (DHBE). While basal expression of E-cadherin in DHBE cells was not different from that of NHBE cells (Fig. 1a), DHBE cells showed a significant increase in mRNA expression of typical mesenchymal markers including N-cadherin and vimentin (Fig. 1b,c). The ratio of E-cadherin to N-cadherin, an index of the epithelial phenotype, (Fig. 1d) was lower (but not statistically significant) in DHBE cells than in NHBE cells, suggesting the potential for EMT in COPD epithelial cells.

**Fig. 2:**
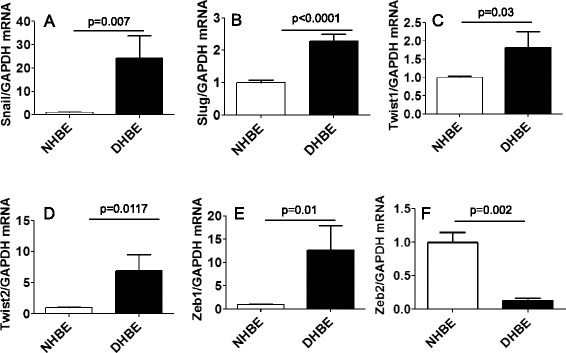
Basal mRNA expression of EMT-related transcription factors, except Zeb2, was higher in DHBE cells. Basal mRNA expression of EMT-related transcription factors in non-COPD (NHBE, open bar, *n* = 3) and COPD (DHBE, closed bar, *n* = 3) human bronchial epithelial cells were analyzed by real-time RT-PCR. Results were normalized to NHBE and expressed as mean (SE) of three independent experiments. Data were analysed by Student *t* test, *p < 0.05, **p < 0.01, ***p < 0.0001, compared with NHBE. (**a**) Snail (**b**) Slug (**c**) Twist1 (**d**) Twist2 (**e**) Zeb1 and (**f**) Zeb2

We next measured changes in the expression of EMT-related transcription factors in NHBE and DHBE cells, including Snail, Snai2 (also called Slug), Twist1, Twist2, and Zeb1. There were significant increases in the expression of these transcription factors in DHBE cells compared to NHBE cells (Fig. 2), findings which are consistent with the hypothesis that the EMT process is more evident in DHBE cells. In contrast, Zeb2 expression was decreased in DHBE cells compared to NHBE cells (Fig. 2f).

**Fig. 3:**
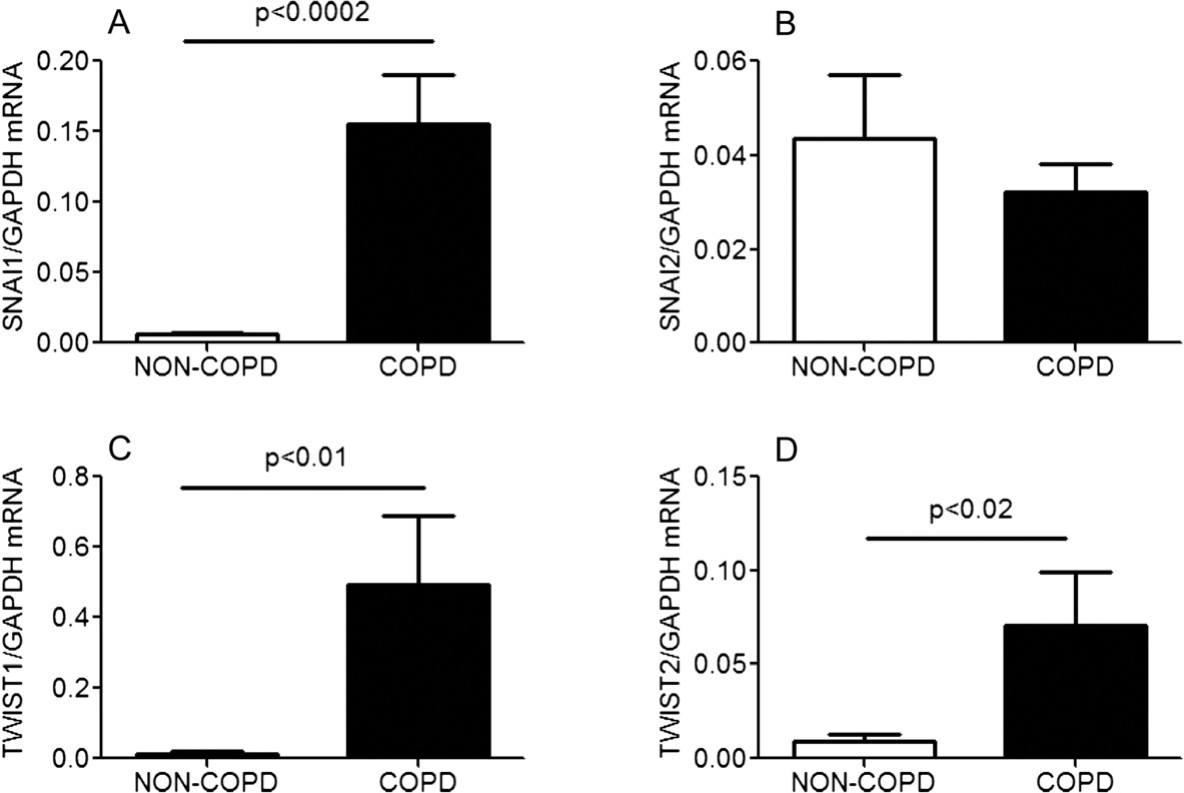
mRNA expression analysis of EMT-related transcription factors in non-COPD and COPD lungs. Basal mRNA expression of EMT-related transcription factors in non-COPD (white bars, *n* = 4) and COPD (black bars, *n* = 4) subjects. Lung tissues from non-COPD and COPD subjects were analyzed by real-time RT-PCR. Results were normalized to housekeeping gene (GAPDH mRNA) and expressed as mean (SE) of four independent experiments. Data were analysed by unpaired Student’s *t* test, (**a**) Snail (**b**) Snai2 (**c**) Twist1 (**d**) Twist2. Non-COPD subjects are non smokers

To determine if the results presented above in bronchial epithelial cells of control (non-COPD) subjects and COPD subjects is also reflective of changes within the lung, we evaluated the expression of these transcription factors in RNA derived from resected lung tissue. As shown in Fig. 3, there was a significant increase in the mRNA expression of the EMT-related transcription factors Snai1 (Fig. 3a), Twist1 (Fig. 3c) and Twist2 (Fig. 3d) in tissue from COPD patients compared to tissue from normal subjects. However, there was no difference in the expression level of Snai2 mRNA (Fig. 3b) between non-COPD and COPD lung.

**Fig. 4:**
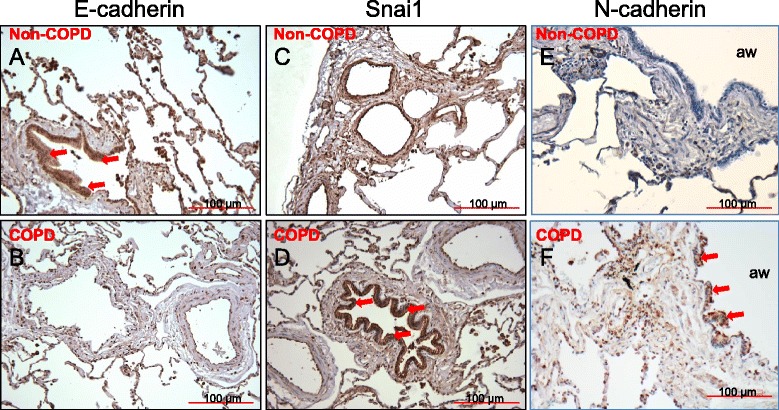
Snail1 and E-cadherin protein expression in non-COPD and COPD lungs. Immunohistochemical localization of EMT markers in the airway epithelium of non-COPD (**a**, **c**, **e**) and COPD (**b**, **d**, **f**) lungs (*n* = 3). Immunostaining for E-cadherin (**a**, **b**) and Snail1 transcription factor (**c**, **d**) and N-cadherin (**e**, **f**). The dark brown color is indicative of strong expression (denoted by red arrows) of E-cadherin in non-COPD (**a**), Snail1 in COPD (**d**) and N-cadherin in COPD (**f**) lung. Negative control was stained with isotype-matched control antibody (data not shown). Non-COPD subjects are non smokers. aw: airway lumen

We also evaluated EMT markers in histological sections of non-COPD (Control) (Fig. 4a,c) and COPD (Fig. 4b,d) lungs. In agreement with mRNA expression, we observed enhanced Snail1 (Fig. 4d) and N-cadherin (Fig. 4f) protein–positive cells in COPD lungs whereas E-cadherin was markedly decreased in COPD (Fig. 4b) compared to non-COPD lungs. Taken together these results corroborate the data obtained from the cell culture model, and support a role for EMT in COPD pathogenesis.

### Basal expression of HGF in non-COPD and COPD lung fibroblasts

**Fig. 5:**
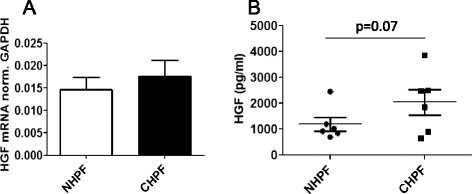
Basal expression of HGF in non-COPD (NHPF) and COPD (CHPF) lung fibroblasts. mRNA expression (**a**) and the protein secretion (**b**) of hepatocyte growth factor in conditioned medium of normal (NHPF) and COPD (CHPF) lung fibroblasts. Results were expressed as mean (SE) of six independent experiments. Data were analysed by unpaired Student’s *t* test, Non-COPD subjects are non smokers

Using RT-PCR, the mRNA expression level of HGF was measured in normal and COPD lung fibroblasts. No significant difference in mRNA expression was found (Fig. 5a). Nor did we find a significant differences between normal and COPD lung fibroblasts in HGF protein secretion in the culture supernatants, although there was a tendency to an increase in COPD fibroblasts (Fig. 5b).

### CM derived from NHLF increased mRNA expression of vimentin in NHBE and DHBE, and that of N-cadherin in DHBE

**Fig. 6:**
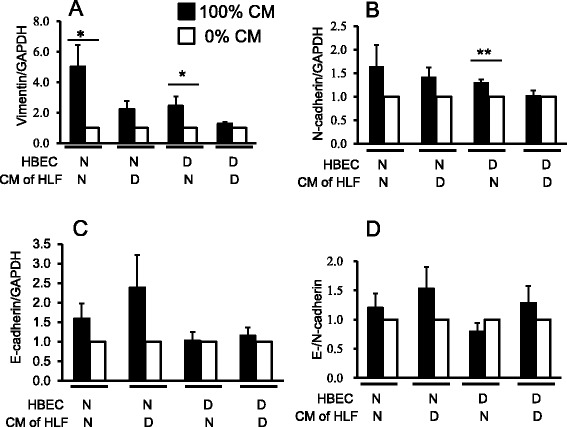
mRNA expression of EMT markers in HBE cells after 3-day treatment with CM from HLF. HBE cells from non-COPD (N) or COPD patients (D) were stimulated with 100 % CM (black bars), or without CM (white bars) collected from human lung fibroblasts (HLF) derived from non-smokers (N) or COPD subjects (D) for 3 days and total RNA was extracted. The mRNA expression of EMT markers were determined by real-time RT-PCR and normalized to GAPDH mRNA expression in the same origin of HBE cells. (**a**) E-cadherin, (**b**) N-cadherin, (**c**) vimentin, and (**d**) the ratio of E-/N-cadherin were calculated. Data were analysed and expressed as mean (SE) of from six to nine independent experiments by one-way ANOVA with Tukey’s multiple comparison test, *p < 0.05, **p < 0.01, compared between the indicated groups

Although the above findings indicate the evidence of EMT in COPD epithelial cells and lungs, it is important to further dissect the contribution of fibroblast-epithelial cell interactions in the EMT process. To this end, we investigated whether soluble mediators secreted by lung fibroblasts would modulate the phenotypic properties of bronchial epithelial cells derived from normal (non-COPD) subjects and COPD patients. NHBE cells and DHBE cells were treated with or without CM derived from NHLF or DHLF for 3 days. In both NHBE and DHBE cells following the 3-day stimulation with CM derived from NHLF, the expression of vimentin mRNA was significantly increased compared to control, and the induction was higher in NHBE cells than in DHBE cells (Fig. 6a). Only in DHBE cells did stimulation with CM derived from NHLF increase N-cadherin mRNA expression (Fig. 6b). Stimulation of HBE cells with CM derived from DHLF did not affect the mRNA expression of N-cadherin or vimentin. Also, there was no significant difference in the expression level of E-cadherin mRNA (Fig. 6c) or E-cadherin/N-cadherin ratio (Fig. 6d) in epithelial cells stimulated with CM derived from NHLF or DHLF. These results suggest that mediators secreted from lung fibroblasts of non-COPD subjects influence EMT events in epithelial cells.

### CM from DHLF increased protein expression of vimentin as well as E-cadherin in NHBE cells

**Fig. 7:**
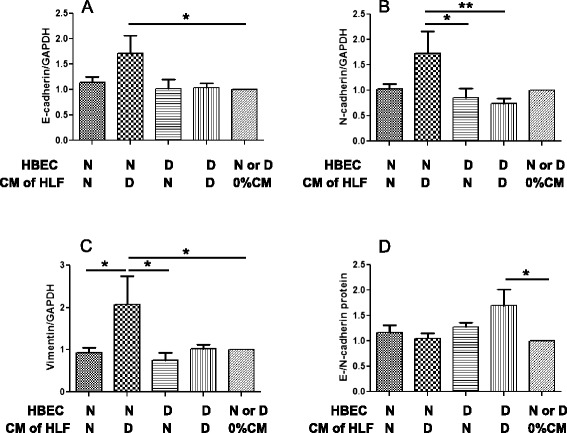
Effects of CM from HLF on protein expression of EMT markers in HBE cells. HBE cells from normal subjects (N) or COPD patients (D) were lysed after stimulation with or without 100 % CM collected from human lung fibroblasts of N or D for 5 days. Total protein was prepared analysed by western blotting, using 40 μg of protein per lane. The membranes were probed with anti-E-cadherin, anti-N-cadherin, anti-vimentin, or anti-GAPDH antibodies. The intensities of the bands for target protein were normalized to the corresponding GAPDH bands for the each treated cells. (**a**) E-cadherin, (**b**) N-cadherin, (**c**) vimentin, and (**d**) the ratio of E-/N-cadherin were calculated. Data were analysed and expressed as mean (SE) of from seven to ten independent experiments by one-way ANOVA with Tukey’s multiple comparison test, *p < 0.05, **p < 0.01, compared between the indicated groups

Based on the mRNA results above, we next investigated the effect of CM on protein expression of EMT markers. Treatment with CM from DHLF significantly increased the protein expression of E-cadherin and N-cadherin in NHBE cells compared to NHLF (Fig. 7a,b). Vimentin protein expression in NHBE cells was increased significantly by CM from DHLF (Fig. 6c), whereas CM from NHLF had no effect. The above data (Figs. 6 and 7) suggest a divergence between protein expression of EMT markers and the corresponding mRNA expression stimulated by NHLF and DHLF CM in NHBE and DHBE cells.

### CM from NHLF increased the mRNA expression of some EMT-related transcription factors in HBE cells

**Fig. 8:**
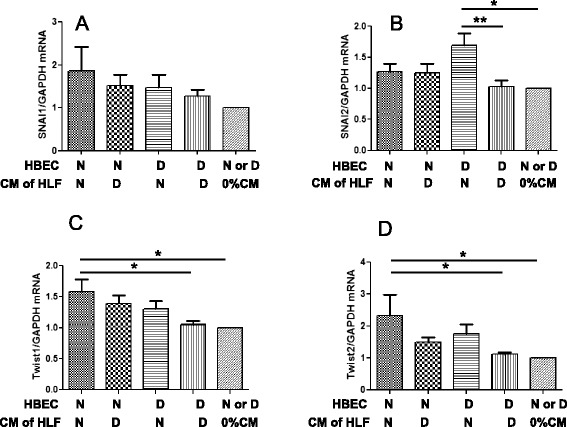
mRNA expression of EMT-related transcription factors in HBE cells after 3-day treatment with CM from HLF. HBE cells from normal subjects (N) or COPD patients (D) were stimulated with or without 100 % CM collected from human lung fibroblasts of N or D for 3 days and total RNA was extracted. The mRNA expressions of EMT-related transcription factors were determined by real-time RT-PCR and normalized to GAPDH mRNA expression in the same origin of HBE cells. (**a**) Snail, (**b**) Slug, (**c**) Twist1, (**d**) Twist2, and (**e**) Zeb1 were calculated. Data were analysed and expressed as mean (SE) of from six to nine independent experiments by one-way ANOVA with Tukey’s multiple comparison test, *p < 0.05, **p < 0.01, compared between the indicated groups

Given the effect of CM on EMT markers in epithelial cells (Figs. 6 and 7), we next determined the changes in the expression of EMT-related transcription factors. CM from NHLF or DHLF did not alter the mRNA expression of Snai1 transcription factor in epithelial cells (Fig. 8a). While CM obtained from NHLF increased the mRNA expression of Snai2 in DHBE cells (Fig. 8b), CM derived from DHLF had no effect. CM from NHLF, but not from DHLF, significantly increased the mRNA expression of Twist1 and Twist2 in NHBE cells (Fig. 8c,d). Collectively, these data may reflect differences in functional phenotypes related to repair and remodeling of fibroblasts derived from non-COPD and COPD subjects in driving the EMT process.

## Discussion

COPD is a complex disease caused primarily by cigarette smoke exposure. The pathogenic processes associated with COPD include α_1_-anti-trypsin deficiency, oxidative stress, chronic inflammation, aberrant repair processes and small airway fibrosis. Recently, EMT has been considered as another causative mechanism implicated in the small airway fibrosis in COPD [4, 5, 14, 15]. In this regard, EMT may contribute to the myofibroblast accumulation associated with fibrotic lesions. Here we demonstrate that under basal culture conditions mRNA expression of mesenchymal markers and EMT-related transcriptional factors are upregulated in DHBE cells compared to NHBE cells. Additionally, our studies demonstrate increased expression of EMT markers and EMT-related transcription factors in COPD lungs compared to non-COPD tissue. Milara et al. [14] recently reported that mesenchymal markers such as vimentin and α-smooth muscle actin (α-SMA) were upregulated in HBEC from smokers and patients with COPD compared to those from non-smokers while epithelial markers such as E-cadherin and ZO-1 were downregulated, findings consistent with our study. To the best of our knowledge, we are the first to show that mRNA expression of EMT-related transcription factors are upregulated in DHBE cells and COPD lungs compared to NHBE cells and non-COPD tissue. These results suggest that DHBE cells have already acquired the phenotype consistent with the EMT process. Furthermore, elevation of EMT markers and transcription factors in DHBE cells relative to normal epithelial cells suggest that COPD epithelial cells are capable of maintaining the EMT phenotype in culture conditions. These data indicate that acquired changes of epithelial phenotypes can contribute to the pathogenesis of COPD.

A defining feature of EMT is a reduction in E-cadherin levels and a concomitant production of N-cadherin. While our immunostaining data revealed evidence of a decrease in E-cadherin and an increase in N-cadherin expression in COPD patients, we did not detect a significant change in E-cadherin gene expression in COPD epithelial cells or in the conditioned medium treated epithelial cells. Based on these observations it could be suggested that epithelial cells in these COPD subjects are in the process of undergoing an EMT program or have attained ‘hybrid states’, with features of both epithelial and mesenchymal cells. Our findings are in agreement with the studies of Sohal et al. [4], who reported that cells in the reticular basement membrane were positive for mesenchymal markers and an epithelial marker in COPD subjects. It has been claimed that cells expressing mesenchymal and epithelial features are migratory, invade the basement membrane, accumulate in the interstitium, eventually lose their epithelial properties and attain a complete fibroblastic phenotype [3].

Mechanisms which drive the EMT process in COPD are largely unknown. Epithelial cells are at the interface with the external environment, and interact extensively with both immune and structural cells such as fibroblasts. Fibroblasts, in addition to being the key cell involved in structure and repair, have enormous synthetic capabilities. COPD fibroblasts exhibit alterations in proliferation, repair and inflammatory cytokine secretion, making it possible that factors released by fibroblasts can influence overlying epithelial cells, thereby altering the EMT process [16]. To address this possibility, we utilized cell culture media (CM) collected from normal and COPD fibroblasts and exposed normal and COPD epithelial cells to this media. Our results demonstrate that CM from NHLF increases mesenchymal markers at mRNA levels in NHBE cells. While the identity of the factor secreted by the fibroblasts is not yet known, a candidate protein is hepatocyte growth factor (HGF). HGF secretion by pulmonary fibroblasts is crucial to an effective epithelial repair after lung injury and can drive the EMT process. HGF secretion by human fibroblasts from emphysematous lung is lower even after stimulation with interleukin-1β or prostaglandin E_2_ compared to control fibroblasts [17]. Thus, low HGF production in COPD fibroblasts, as reported earlier by Plantier et al. [17], may explain why these cells are less capable of inducing EMT compared to normal fibroblasts.

Our results showed no significant change in the expression of HGF between normal (NHLF) and diseased lung fibroblasts (DHLF), although the protein expression data showed a tendency to an increase. The differences in HGF expression between our study and that of Plantier et al. [17] might be due to differences in disease severity. Whereas fibroblasts used in our study were obtained from patients with mild to moderate COPD, cells used in the study of Plantier et al. [17] were from patients with more severe disease who were undergoing lung volume reduction surgery. As fibroblasts secrete soluble factors which act in a paracrine manner targeting epithelial repair and maintenance, alterations in HGF production due to cigarette smoking may well have implications for wound repair functions of fibroblasts in COPD.

Multiple transcription factors, including Twist, Snail, Slug, and Zeb orchestrate the EMT and the migratory processes during embryogenesis [18–22]. Of these, Twist is particularly important, as it can bind to the E-cadherin promoter and represses gene transcription of E-cadherin, a protein required in cell-cell contact and one that is typically down-modulated during EMT. In the lung, Pozharskay et al. first demonstrated the upregulation of Twist protein expression in a murine model of virus-induced lung fibrosis and in idiopathic pulmonary fibrosis (IPF) lung tissue [23]. However, there is no information on the expression of Twist in COPD or influence by epithelial cells. Therefore, we evaluated whether CM could alter the expression of EMT-associated transcription factors in COPD cells. Our data show that only CM from NHLF significantly affected Twist1 and Twist2 expression in NHBE cells. While the functional significance of this observation in the context of COPD is not clear, it is possible that Twist2 contributes to the maintenance of epithelial cell characteristics [21] or participates as an anti-apoptotic factor [22]. Further studies are needed to characterize the functional significance of Twist in COPD. Regardless, our studies suggest that CM from NHLF-induced Twist activation might represent an important mechanism for EMT in the normal airway epithelial repair.

Interestingly, NHLF-derived CM was also more effective than DHLF-derived CM at inducing Slug mRNA in DHBE cells. Concomitant with the induction of EMT, Slug has a critical role in re-epithelialization of cutaneous wounds in mice, a process that involves a transient loss of epithelial polarity without full acquisition of mesenchymal characteristics. Such partial EMT processes occur during some types of tubulogenesis (e.g., in mammary gland development). Slug is also required for survival during partial EMT [24]. In our study, CM from normal HLF increased the expression of Slug in DHBE cells, which may mean that normal lung fibroblasts are capable of promoting cell survival and thus contribute to the normal repair of the injured DHBE cells. We also examined the expression of Zeb1 and Zeb2 mRNA in DHBE cells. While Zeb1 expression was upregulated in DHBE cells, Zeb2 mRNA expression was decreased in DHBE compared to NHBE. It is known that E-cadherin expression is regulated by a family of transcriptional factors including Snail, Slug, Twist, Zeb1, and Zeb2, and these proteins are, in turn, regulated by growth factors such as PDGF, TGF-β, and Wnt proteins. Both Zebs suppress E-cadherin expression. However, it is not clear if Zeb1 has a predominant role over Zeb2 or *vice versa* in suppressing E-cadherin expression during EMT. It is likely that both Zebs contribute to EMT, along with involvement of other EMT-related transcription factors. Further studies are needed to address their respective roles using molecular strategies designed to target simultaneously both Zebs and/or other transcription factors. Our data also raise the question whether different repressors participate in silencing of E-cadherin at defined stages of EMT program in COPD. Together, our results illustrate the complexity of E-cadherin expression and demonstrate that a mechanism that fully defines its expression in COPD has yet to be described.

We also found that protein expression of both epithelial and mesenchymal markers were increased only in NHBE cells treated with CM from DHLF. A possible explanation is that because the basal expression of EMT markers is higher in COPD epithelial cells, these cells are refractory to subsequent treatment with CM and cannot further upregulate the expression of EMT markers. The increased levels of EMT markers in diseased epithelial cells derived from COPD subjects may be due to cigarette smoking, which has been shown to promote EMT in epithelial cells and emphasizes the importance of environmental factor in the pathogenesis of chronic inflammatory diseases, including COPD [5, 6]. The above changes in EMT protein markers seem to be contradictory with data obtained for mRNA expression where NHLF-derived CM upregulated EMT-associated transcription factors in DHBE cells. The molecular mechanisms responsible for the differential actions of CM from normal vs. COPD lung fibroblasts on protein and mRNA expression are not clearly understood.

In summary, our work reveals increases in EMT-associated transcription factors and mesenchymal markers in bronchial epithelial cells derived from COPD subjects, demonstrating that these cells are undergoing EMT process. The fact that only normal bronchial epithelial cells responded to cell culture conditioned-medium derived from COPD lung fibroblasts suggest that environmental factors (e.g., cigarette smoke) may play an important role in the development of EMT. Although recent reports have indicated that human bronchial epithelial cells from COPD patients compared to non-smokers, undergo EMT, the hypothesis that epithelial trans-differentiation during tissue repair in COPD may be induced through the communication with fibroblasts, independent of inflammatory cells, was not considered in previous studies [4, 5, 14]. The epithelium-fibroblast interaction is important in normal lung development, where a fibroblast-secreted mediator affected epithelial maturation and function [25]. During an injury-repair response secreted mediators are required for epithelial regeneration. In the case of anomalous repair the balance is tipped in the direction of a fibrotic growth, where interruption of the normal intercellular relationships, principally between epithelial cells and fibroblasts, occurs. Whether these intercellular contacts transfer inductive messages associated with switching on EMT is unknown. Here, we investigated whether fibroblasts in COPD subjects contribute to the abnormal function of epithelial cells, and the effect of mediators secreted by human lung fibroblasts from normal and COPD subjects on phenotypic changes in normal and diseased bronchial epithelial cells. We demonstrate that cell cultured conditioned medium from COPD fibroblasts (DHLF-CM) promoted EMT in normal human bronchial epithelial cells (NHBE), suggesting that interactions between fibroblast and epithelial cells may play an important role in the EMT process in COPD. Whereas mediators secreted by infiltrating inflammatory cells, such as lymphocytes, platelets or macrophages, upregulate EMT-related transcription factors [25–29], and may be critical to establishment of repair response by epithelial cells and fibroblasts, our data are novel as they demonstrate that the interrelationship between epithelial and fibroblast cells is sufficient to induce EMT process in the absence of blood-borne cells or their secreted mediators. Our findings also indicate the possibility that fibroblasts are involved in the control of EMT via soluble mediators, a finding which merits further investigation. The finding that structural cells may be involved directly or indirectly in promoting EMT further establishes the importance of these cells in COPD pathogenesis.

## Abbreviations

CM: Conditioned medium
COPD: Chronic obstructive pulmonary disease
ECM: Extracellular matrix
EMT: Epithelial-mesenchymal transition
HBE: Human bronchial epithelial cell
HLF: Human lung fibroblast
NHBE: Normal human bronchial epithelial cells
DHBE: COPD bronchial epithelial cells
NHLF: Normal hulan lung fibroblasts
DHLF: COPD lung fibroblasts
HGF: Hepatocyte growth factor.
